# Frailty as a Risk Factor for Depression after COVID-19 Hospital Admission

**DOI:** 10.3390/geriatrics9040097

**Published:** 2024-07-22

**Authors:** Isabel María Soler-Moratalla, Sergio Salmerón, Silvia Lozoya-Moreno, Ana María Hermosilla-Pasamar, Antonio Henández-Martínez, Julián Solís-García del Pozo, Margarita Escribano-Talaya, Maria Antonia Font-Payeras, Francisco García-Alcaraz

**Affiliations:** 1 Department of Geriatrics, General Hospital of Villarrobledo, 02600 Villarrobledo, Spain; isabelsolermoratalla@hotmail.com; 2 San Vicente de Paúl Nursing Home, Diputación de Albacete, 02001 Albacete, Spain; 3 Department of Geriatrics, Albacete University Hospital Complex, 02008 Albacete, Spain; slozoyamoreno@gmail.com; 4 Clinical Psychology, Albacete University Hospital Complex, 02008 Albacete, Spain; ahermosilla@hotmail.com; 5 Faculty of Nursing of Ciudad Real, Castilla-La Mancha University, 13003 Ciudad Real, Spain; antonio.hmartinez@uclm.es; 6 Deparment of Internal Medicine, Albacete University Hospital Complex, 02008 Albacete, Spain; julianeloysolis@gmail.com; 7 Deparment of Radiology, Hospital of Villarrobledo, 02600 Villarrobledo, Spain; margaritae@sescam.jccm.es; 8 Clinical Psichology, UCA Ponent Ibsalut, 07013 Mallorca, Spain; fontpayeras@yahoo.es; 9 Nursing School, Castilla-La Mancha University, 02071 Albacete, Spain; fgarciaalcaraz@gmail.com

**Keywords:** COVID-19, depression, frailty, mental health, SARS-CoV-2, pandemic, vitamin D

## Abstract

Background: This work aims to establish the relationship between depression and epidemiological or imaging variables, frailty, and cognitive status in patients who suffered hospital admission for COVID-19. Methods: A longitudinal observational study investigated 72 patients admitted for COVID-19 to a hospital in Spain. Patients were evaluated at discharge and six months later. Clinical, analytical, and imaging variables were collected. A neurocognitive, nutritional, and frailty (FRAIL scale) assessment of the included patients was carried out. The risk of depression was considered for a result above 5 points on the PHQ-9 scale. Results: The variables that were significantly related to the risk of depression 6 months after admission for COVID-19 were frailty (*p* = 0.006 for pre-frail and *p* = 0.001 for frail), small-vessel vascular disease in imaging tests (*p* = 0.033), vitamin D level (*p* = 0.006), and taking antidepressants (*p* = 0.011). Factors that were negatively associated with the presence of depression 6 months after discharge were a higher score on the CAMCOG cognitive scale (*p* = 0.041) and older age (*p* = 0.006). Conclusions: Frailty worsened the score on the PHQ-9 depression scale in patients who required hospital admission for SARS-CoV-2 infection. It is important to implement prevention measures both for frailty and depression in these patients.

## 1. Introduction

The SARS-CoV-2 infection has had a great impact on the healthcare of the population of different countries. This impact is usually measured in mortality and the number of hospital admissions and has tested the solidity of the different health systems in the world. Most European countries have felt the effects of this pandemic with great intensity despite having advanced health systems [1,2].

However, COVID-19 has other health implications for the affected population, mainly in the population that has suffered an admission due to a severe case of COVID-19. Among these implications are the consequences for mental health that, however, are often overlooked by the clinician. There are several works and systematic reviews that have linked the appearance of mental health problems (such as anxiety and depression) to COVID-19, both in patients [3,4] and in health workers [5]. Frailty status before the COVID-19 outbreak was associated with higher odds of persistent and incident common mental disorders in older adults during the pandemic’s first wave [6]. In the case of admission due to COVID-19, an increase in fragility has also been detected 12 weeks after admission [7]. The increased prevalence of depressive symptoms associated with the stay-at-home order could also have influenced the increase in the prevalence of social frailty and changes in quality of life, lifestyle habits, and psychosocial status in older adults during the COVID-19 pandemic, including in those elderly who had not been infected with COVID-19, where up to 56% reported substantial lifestyle modifications [8,9].

The term “depression” encompasses a set of disorders characterized by an altered mood with sadness that significantly interferes with daily life due to both its intensity and duration [10]. They can also be accompanied by reduced interest and pleasure in carrying out activities [11]. It has been estimated that the prevalence of depression worldwide is 5.0% in the adult population [12]. Depressive disorders continue to be a problem worldwide with an increase in age-standardized disability-adjusted life-years (DALY) rates 16.4% (95% UI 11.9–21.3) [13]. In Spain, a prevalence of 5.4% has been estimated, with differences between women and men (7.1% vs. 3.5%, respectively) [14].

Although it has been confirmed that COVID-19 can be a precipitating factor for mood alterations [15,16], the relationship between epidemiological variables, the presence of frailty, biological variables, treatment received, or brain imaging and the development of these disorders is not well defined. In the present study, we aim to establish if there is a relationship between depression and these variables in patients who have been admitted for COVID-19 in a hospital in Castilla-La Mancha (Spain).

## 2. Materials and Methods

### 2.1. Population and Sample

This longitudinal observational study includes patients admitted for COVID-19 at the Villarrobledo Hospital (Spain) in 2020. It is a 103-bed first-level hospital located in an area with a predominantly rural population and serving an area of approximately 65,000 inhabitants. Inclusion criteria: patients admitted with a microbiologically confirmed diagnosis of COVID-19 by polymerase chain reaction (PCR) for nasopharyngeal exudate or another valid sample (obtained by bronchoscopy or other instrumental methods); patients who, at the time of the assessment, were either cured (proven by negative PCR) or had been discharged from the hospital at least six weeks prior; and patients who were at least 55 years old. Exclusion criteria: patients with a previous diagnosis of cognitive impairment or psychiatric pathology; patients with any medical pathology that made them unable to perform neuropsychological tests such as aphasia, severe visual deficit, motor paralysis, or severe hearing loss; and patients who refused to sign the informed consent. Patients taking antidepressant medication for reasons other than the presence of mood disorders, such as chronic or neuropathic pain, were allowed in the study. Patients had to meet all three inclusion and none of the exclusion criteria to be eligible for the study.

### 2.2. Variables

Variables collected were:

- Demographic variables: age, sex, and educational level;
- Clinical service in which the patients were hospitalized;
- Clinical data: days from the onset of symptoms to admission, symptoms, comorbidity, and Charlson index; medication prescribed during admission and at hospital discharge; minimum oxygen saturation on admission, maximum FiO2 during admission, and oxygen saturation at evaluation visits. Frailty assessment was carried out using the FRAIL scale [17], which resulted in classifying the patients as either robust, pre-fragile, or fragile (independent variable);
- Neuropsychological assessment: CAMCOG (Cambridge Cognition Examination) scales [18] and the INECO Frontal Screening [19] for dementia, and PHQ-9 scale (Depression Patient Health Questionnaire) for depression (dependent variable) [20]. The PHQ-9 scale consists of 9 items, each with a score between 0 and 3, and a possible total score of 27. A patient’s total score can be interpreted as follows: no depression (<5 points); mild depression (5–9 points); moderate depression (10–14 points); moderately severe depression (15–19 points); and severe depression (≥20) [21];
- Nutritional assessment: CONUT (controlling nutritional status) [22];
- Analytical findings during admission (including leukocytes, C-reactive protein, ferritin, D-dimer, and interleukin-6) and after admission (including creatinine, folic acid, vitamin B12, thyroid hormones, cholesterol, albumin, lymphocytes, blood group, and vitamin D);
- Magnetic resonance imaging (MRI): the presence of cerebral atrophy, subjective white matter alterations of cerebral small-vessel disease (CSVD), large-vessel stroke, hemorrhage, space-occupying lesion, and normal-pressure hydrocephalus.

### 2.3. Interventionism and Follow-Up

Patients were evaluated in two visits. The first was 14 days after hospital discharge (in order to avoid COVID-19 infections), when they were informed of the study and signed the informed consent. At this time, the FRAIL scale, CONUT, and the first neuropsychological assessment were performed. An analytical extraction and oxygen saturation measurement were carried out along with these scales. An MRI was then requested, which was evaluated on a second visit, in which the cognitive, nutritional, and depression scales were also repeated, as well as a second oxygen saturation measurement. We performed this second neuropsychological assessment at 6 months because the Spanish Society of Geriatrics and Gerontology in its “Consensus document on mild cognitive impairment in older adults” recommends a second neuropsychological re-evaluation, after the first visit, at 6 months.

### 2.4. Statistical Analysis

For the statistical analysis, a descriptive study of the variables collected was carried out using frequencies for qualitative variables and measures of central tendency (mean or median) and dispersion (standard deviation or interquartile range) in the case of quantitative variables. Next, bivariate and multivariate analyses was carried out on the risk of depression (total score ≥ 5 on the PHQ-9 scale) and the different variables collected: sociodemographic, clinical, analytical, and imaging tests using binary logistic regression. Crude and adjusted odds ratios (ORs) were estimated with their respective 95% confidence intervals (95% CIs). Finally, a predictive model of the risk of depression 6 months after the episode of hospital admission for COVID-19 was created, and its area under the ROC (receiver operating characteristic) curve (AUC) was estimated. This model was constructed by using backward elimination (RV in SPSS statistical package). To create the model, all those sociodemographic and clinical variables (pathologies, laboratory tests, and imaging tests) that could influence the risk of depression were included: sex, age, education level, hypertension, DM2, COPD, asthma, OSAHS, tobacco, frailty, BMI, Charlson comorbidity index, days of admission, atrophy on MRI, cerebral small-vessel disease, CAMCOG, vitamin D, CONUT, and frontal screening. The automatic selection model was used with PIN values (0.05), POUT values (0.10), LCON (0), and BCON (0.001). PIN specifies the minimum probability that a variable can have of entering the analysis and POUT specifies the maximum probability that a variable can have and not be eliminated from the model. BCON specifies the change in parameter estimates to terminate the iteration and LCON specifies the percentage change in the log likelihood ratio to terminate the iteration. In order to assess the prediction qualitatively, we used Swets’s criteria, for which the value ranges are 0.5–0.6 (bad), 0.6–0.7 (poor), 0.7–0.8 (satisfactory), 0.8–0.9 (good), and 0.9–1.0 (excellent) [23]. The SPSS^®^ version 28.0 statistical package was used for this analysis.

### 2.5. Ethical Considerations

Our study was conducted with the utmost respect for ethical considerations. All patients provided written, informed consent after being fully briefed on the study objectives and procedures. The study protocol was designed in accordance with the Declaration of Helsinki and was approved by the Drug Research Ethics Committee of Albacete for studies involving humans (protocol code 2020/05/057, approved on 28 July 2020). 

## 3. Results

### 3.1. Demographics

Seventy-two patients were evaluated both at discharge and 6 months after the acute episode of COVID-19. By gender, 47 (65.3%) were men, and 25 (34.5%) were women. The mean patient was 69.06 years old (SD = 8.70 years old). More than 70% of the patients had an elementary or lower educational level, 26.4% suffered from diabetes, 26.4% were classified as fragile, 55.6% as pre-frail, and 18.1% as robust. The most important characteristics of the studied cohort are collected in Table 1. 

### 3.2. Risk of Depression at Hospital Discharge

Table 2 shows the bivariate and multivariate analyses between subjects with a score on the PHQ-9 scale below 5 and those with a score of at least 5 in the assessments carried out at hospital discharge:

- Bivariate analysis at hospital discharge: It could be observed that frailty status was associated with a worse PHQ-9 score with an OR of 11.92 (95% CI 1.99–71.41; *p* = 0.007) compared to robust patients. There was also a significant association with the body mass index (OR 1.21; 95% CI 1.04–1.41; *p* = 0.007).
- Multivariate analysis at hospital discharge: The status of pre-frail (adjusted odds ratio (aOR) 23.25; 95% CI 2.36–229.20; *p* = 0.007) and frail (aOR 125.23; 95% CI 6.34–2476.11; *p* = 0.002) was related to the risk of depression. The age was also significant (aOR 0.88; 95% CI 0.78–0.99; *p* = 0.027).

**Table 2:** Factors associated with the risk of depression (PHQ-9 ≥ 5) at hospital discharge. Bivariate and multivariate analyses.

Variable	PHQ-9 Normal (*n* = 40) *n* (%)	PHQ-9 ≥ 5 (*n* = 32) *n* (%)	Bivariate Analysis		Multivariate Analysis	
			OR (CI 95%)	*p*	aOR (CI 95%)	*p*
Age (mean ± SD)	69.98 ± 7.57	67.91 ± 9.95	0.97 (0.92–1.03)	0.320	0.88 (0.78–0.99)	0.027
Sex				0.347		
Men	28 (70.0%)	19 (59.4%)	1			
Women	12 (30.0%)	13 (40.6%)	1.60 (0.60–4.24)			
Hypertension	18 (45.0%)	18 (56.3%)	1.57 (0.62–4.01)	0.344		
DM2 ^1^	9 (22.5%)	10 (31.3%)	1.57 (0.55–4.49)	0.404		
COPD ^2^	6 (15.0%)	0 (0.0%)		0.999		
Asthma	6 (15.0%)	2 (6.3%)	0.38 (0.07–2.02)	0.287 *		
OSAHS ^3^	4 (10.0%)	3 (9.4%)	0.93 (0.19–4.50)	0.929 *		
Tobacco	4 (10.0%)	3 (9.4%)	0.93 (0.19–4.50)	0.929 *		
FRAIL scale				0.011		0.006
Robust	11 (27.5%)	2 (6.3%)	1		1	
Pre-frail	23 (57.5%)	17 (53.1%)	4.07 (0.80–20.79)	0.092	23.25 (2.36–229.20)	0.007
Frail	6 (15.0%)	13 (40.6%)	11.92 (1.99–71.41)	0.007 *	125.23 (6.34–2476.11)	0.002
Education level				0.791		
Not schooled	10 (25.0%)	5 (15.6%)	1			
Children’s school	9 (22.5%)	7 (21.9%)	1.56 (0.36–6.69)	0.553		
Elementary	9 (22.5%)	11 (34.4%)	2.44 (0.61–9.80)	0.207		
High school	9 (22.5%)	7 (21.9%)	1.56 (0.36–6.69)	0.553		
Higher education	3 (7.5%)	2 (6.3%)	1.33 (0.17–10.74)	0.787		
Atrophy on MRI ^4^	27 (71.1%)	19 (61.3%)	0.64 (0.24–1.77)	0.392		
Antidepressants	2 (5.0%)	4 (12.5%)	2.71 (0.46–15.87)	0.396 *		
CSVD ^5^ on MRI	17 (44.7%)	11 (35.5%)	0.68 (0.26–1.80)	0.436		
Days of admission	9.45 ± 7.05	10.63 ± 6.90	1.03 (0.96–1.10)	0.480		
(mean ± SD)						
Charlson index	1.48 ±1.49	1.44 ± 1.59	0.98 (0.72–1.34)	0.918		
(mean ± SD)						
BMI ^6^	28.44 ± 2.86	30.76 ± 4.20	1.21 (1.04–1.41)	0.007	1.21 (1.01–1.44)	0.035
(mean ± SD)						
CAMCOG ^7^ first visit	88.10 ± 10.69	86.45 ± 11.57	0.99 (0.95–1.03)	0.533		
(mean ± SD)						
Vitamin D level ng/mL	23.55 ± 13.03	24.17 ± 14.27	2.71 (0.46–15.87)	0.850	1.05 (0.99–1.10)	0.109
(mean ± SD)						
CONUT ^8^ admission	4.54 ± 2.33	3.70 ± 2.15	0.83 (0.65–1.06)	0.131	0.66 (0.44–0.97)	0.035
(mean ± SD)						
Frontal screening	21.39 ± 4.08	20.84 ± 4.48	0.97 (0.87–1.08)	0.592	0.83 (0.77–1.04)	0.104
(mean ± SD)						
first visit						

Qualitative variables are expressed as *n* (%) and quantitative variables as mean ± standard deviation. * Fisher’s exact test. ^1^ diabetes mellitus type 2; ^2^ COPD, chronic obstructive pulmonary disease; ^3^ obstructive sleep apnea/hypopnea syndrome; ^4^ magnetic resonance imaging; ^5^ cerebral small-vessel disease magnetic resonance imaging; ^6^ body mass index; ^7^ Cambridge Cognitive Examination; ^8^ controlling nutritional status.

### 3.3. Risk of Depression at 6 Months

The same bivariate and multivariate analyses were conducted to verify the factors associated with the pathological score in the PHQ-9 questionnaire 6 months after the episode of admission for severe COVID-19 (Table 3):

- Bivariate analysis at 6 months: It could be observed that frailty status was associated with a worse PHQ-9 score (OR 13.33; 95% CI 1.43–123.99; *p* = 0.023) compared to robust patients. There was also a significant association with the body mass index (OR 1.16; 95% CI 1.01–1.34; *p* = 0.037).
- Multivariate analysis at 6 months: We observed that frailty measured by the FRAIL scale was associated with the PHQ-9 score at or above 5 at 6 months with an aOR of 383.33 (95% CI 5.04–27,147.57; *p* = 0.006) for the pre-frail state, and an aOR of 83,959.65 (95% CI of 79.86–88,268,687.07; *p* = 0.001) for the frail state. Another variable that was significantly related was the presence of small-vessel cerebrovascular disease (aOR 18.78; 95% CI 1.27–277.76; *p* = 0.033), as well as the level of vitamin D (aOR 1.25; 95% CI 1.06–1.45; *p* = 0.006). Taking antidepressants was related to the presence of a pathological score on the PHQ-9 scale (aOR 112.66; 95% CI 2.99–4246.21; *p* = 0.011), although antidepressants were not used for treating depression since patients not having been diagnosed with depression was a condition to be included in the study. The variables negatively related to the presence of depression at 6 months were age (aOR 0.73; 95% CI 0.59–0.90; *p* = 0.003), the presence of atrophy in brain MRI (aOR 0.16; 95% CI 0.02–1.5; *p* = 0.170), and a higher score on the CAMCOG scale (aOR 0.86; 95% CI 0.75–0.99; *p* = 0.041).

**Table 3:** Factors associated with the risk of depression (PHQ-9 ≥ 5) at 6 months after the episode of admission. Bivariate and multivariate analyses.

Variable	PHQ-9 Normal (*n* = 47) *n* (%)	PHQ-9 ≥ 5 (*n* = 25) *n* (%)	Bivariate Analysis		Multivariate Analysis	
			OR (CI 95%)	*p*	aOR (CI 95%)	*p*
Age (mean ± SD)	69.68 ± 7.85	67.88 ± 10.18	0.98 (0.92–1.03)	0.402	0.73 (0.59–0.90)	0.003
Sex				0.230		
Men	33 (70.2%)	14 (56.0%)	1			
Women	14 (29.8%)	11 (44.0%)	1.85 (0.68–5.07)			
Hypertension	22 (46.8%)	14 (56.0%)	1.45 (0.55–3.84)	0.459		
DM2 ^1^	12 (25.5%)	7 (28.0%)	1.13 (0.38–3.38)	0.821		
COPD ^2^	4 (8.5%)	2 (8.0%)	0.94 (0.16–5.50)	0.941		
Asthma	6 (12.8%)	2 (6.3%)	0.59 (0.11–3.19)	0.544		
OSAHS ^3^	3 (6.4%)	4 (16.0%)	0.59 (0.11–3.19)	0.504		
Tobacco	4 (8.5%)	3 (12.0%)	2.79 (0.57–13.63)	0.204		
FRAIL scale				0.064		0.006
Robust	12 (25.5%)	1 (4.0%)	1		1	
Pre-frail	26 (55.3%)	14 (56.0%)	6.46 (0.76–54.92)	0.088	383.33 (5.04–27,147.57)	0.006
Frail	9 (19.1%)	10 (40.0%)	13.33 (1.43–123.99)	0.023	83,959.65 (79.86–88,268,687.07)	0.001
Education level				0.861		0.197
Not schooled	11 (23.4%)	4 (16.0%)	1		1	
Children’s school	9 (19.1%)	7 (28.0%)	2.13 (0.47–9.70)	0.324	88.11 (1.83–4244.63)	0.023
Elementary	14 (29.8%)	6 (24.0%)	1.18 (0.27–5.24)	0.829	23.56 (0.86–646.67)	0.062
High school	10 (21.3%)	6 (24.0%)	1.65 (0.36–7.60)	0.521	1.49 (0.026–87.02)	0.845
Higher education	3 (6.4%)	2 (8.0%)	1.83 (0.22–15.33)	0.576	18.86 (0.18–1932.85)	0.214
Atrophy on MRI ^4^	32 (69.6%)	14 (60.9%)	0.68 (0.24–1.94)	0.471	0.16 (0.02–1.500)	0.107
Antidepressants	3 (6.4%)	3 (12.0%)	2.00 (0.37–10.73)	0.419	112.66 (2.99–4246.21)	0.011
CSVD ^5^ on MRI	19 (41.3%)	9 (39.1%)	0.91 (0.33–2.54)	0.862	18.78 (1.27–277.76)	0.033
Admission days	10.60 ± 7.92	8.80 ± 4.57	0.96 (0.88–1.04)	0.306	0.77 (0.58–1.01)	0.062
(mean ± SD)						
Charlson index	1.53 ± 1.53	1.32 ± 1.52	0.91 (0.65–1.27)	0.571		
(mean ± SD)						
BMI ^6^	28.78 ± 3.30	30.76 ± 4.06	1.16 (1.01–1.34)	0.037		
(mean ± SD)						
CAMCOG ^7^ follow-up at 6 months	90.40 ±9.87	90.26 ± 10.37	0.99 (0.95–1.03)	0.528	0.86 (0.75–0.99)	0.041
(mean ± SD)						
Vitamin D level ng/mL	22.29 ± 12.22	23.82 ± 13.50	4.29 (0.73–25.28)	0.108	1.25 (1.06–1.45)	0.006
(mean ± SD)						
CONUT ^8^ follow-up at 6 months	0.91 ± 0.95	1.12 ± 1.45	1.17 (0.77–1.79)	0.468		
(mean ± SD)						
Frontal screening follow-up at 6 months	21.69 ± 4.35	21.54 ± 4.47	0.99 (0.88–1.11)	0.888	0.72 (0.52–1.04)	0.053
(mean ± SD)						

^1^ diabetes mellitus type 2; ^2^ COPD, chronic obstructive pulmonary disease; ^3^ obstructive sleep apnea/hypopnea syndrome; ^4^ magnetic resonance imaging; ^5^ cerebral small-vessel disease magnetic resonance imaging; ^6^ body mass index; ^7^ Cambridge Cognitive Examination; ^8^ controlling nutritional status.

### 3.4. Predictive Capacity of Depression

**Figure 1:**
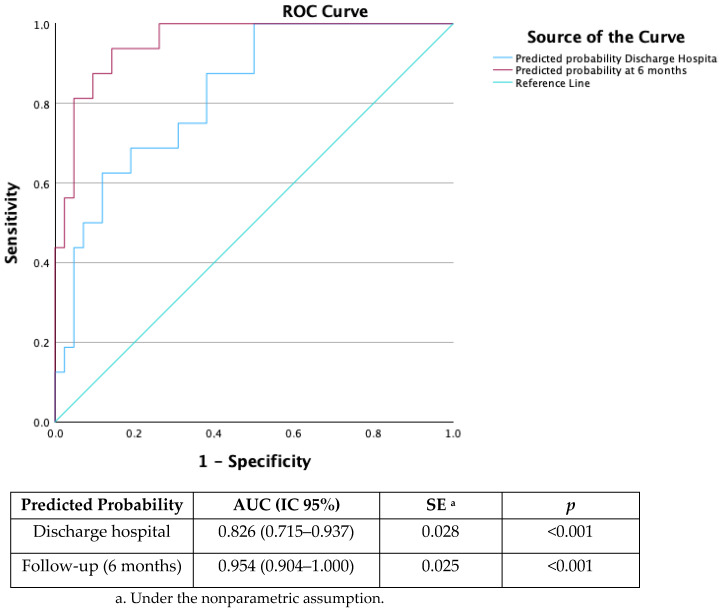
ROC curve of the logistic regression model to predict a PHQ-9 score ≥ 5 at 6 months after hospital discharge.

Finally, the AUC of the ROC of this second logistic regression model was been calculated to evaluate the model’s predictive capacity regarding the presence of at least mild depression after hospital admission for COVID-19 and 6 months after hospital admission. This last model showed an ROC AUC of 0.954 (95% CI of 0.904–1.000; *p* < 0.001) (Figure 1), which is considered excellent following Swets’s criteria.

## 4. Discussion

The present study reveals a series of factors capable of predicting a total score at or above 5 on the PHQ-9 scale 6 months after an episode of COVID-19 that required hospital admission. Furthermore, it shows that some of these factors are already related to the presence of a pathological score from the questionnaire a few days after hospital admission. We consider this result to be very relevant to implementing measures to prevent these patients from developing more severe symptoms of depression.

Among the factors identified, fragility appears prominently. It can be seen that the pre-frail and frail state is associated with a worse result in the depression test both at discharge and at 6 months, and with an OR that is very significant. Frailty has already been associated with other morbid conditions with a three-fold increased adjusted risk of mortality [24] and disability [25]. In a meta-analysis that related frailty to depression [26], frailty increased the risk of incident depression with an OR = 1.90 (95%CI 1.55–2.32, I2 = 0%). Frailty was also significantly associated with elevated risks of depression data from the Psychiatric Genomics Consortium (OR, 1.860; 95% CI, 1.439–2.405; *p* < 0.001) [27]. Furthermore, our study reveals that there is a greater risk in the frail state than in the pre-frail state, which in turn confirms that the higher the score on the FRAIL scale, the more likely it is that the post-COVID-19 patient will present some depressive symptoms.

Our study also provides the novelty of relating this depressive state with the cognitive state assessed by the CAMCOG and the frontal screening scales. Older adults with depression more commonly have cognitive impairment [28], so a key insight from our study is that the higher the CAMCOG scale, the lower the risk of post-COVID-19 depression. It was not possible to demonstrate that the result of this scale predicts a pathological PHQ-9 score at the time of discharge, but it does at 6 months. This finding supports the conclusions of various authors that cognitive reserve may impact neuropsychiatric disorders (like depression) in three ways: by affecting the risk for developing the disorder, in the expression of symptoms within disorders, and in patients’ functional outcomes [29,30]. From a physiological point of view, frailty involves a metabolic state of inflammation, and this inflammation is a risk factor for depression and dementia [31].

Vascular brain lesions related to reversible hemorrhagic encephalopathy syndrome [32] and high thrombotic risk [33] have been described in post-COVID-19 patients. In our study, 38.9% had small-vessel cerebrovascular disease on MRI, which was related to a PHQ-9 score of at least 5. There is already a hypothesis that these cerebrovascular diseases could lead to depression, mainly if they occur in specific brain regions (more frequently associated with the left hemisphere or the posterior right hemisphere) [34].

It has been reported that interferon beta 1b can cause depression as a side effect [35], but this relationship was not found in our study. Age has been indicated as a risk factor for depression in COVID-19 patients over 50 [36], but in our study, we found a protective factor since age presented an OR 0.73 (95% CI: 0.59–0.90; *p* = 0.003) to obtain a PHQ-9 score of at least 5. However, this can be a selection bias as older people survived from COVID-19 are stronger.

Several authors have linked vitamin D deficiency with depression, although they support the acquisition of additional data to better determine the impact of vitamin D in the prevention of depression [37,38]. Our study also supports this relationship between vitamin D deficiency and depression in the post-COVID-19 context. Although we must take into account possible confounding factors that influence this vitamin D deficiency, such as the reduction in outdoor activity due to admission and confinement, the nutritional status that usually worsens as a consequence of admission, and the lack, in Spain, of a national vitamin D fortification policy covering various fluid milk products [39].

Among the study limitations, we highlight that the population comes from a single hospital, therefore constituting a small and non-randomized sample, and the outcomes might not be representative on a broad level. We did not include patients who met the exclusion criteria: patients with a previous diagnosis of cognitive impairment or psychiatric pathology and patients with any medical pathology that made them unable to perform neuropsychological tests. Furthermore, it would have been useful to have a brain MRI prior to or during admission to compare it with the MRI after overcoming COVID-19. The ideal would be to carry out a linear trend analysis, but multinominal analysis implies greater categorization, and together with the low sample size, we do not have enough statistical power to carry out a more precise analysis. If the depression lasts 2 or more years, then it is called chronic depression, so an additional follow-up after 2 years would have been useful for this diagnosis. On the other hand, as a strength, our study stands out for being one of the few that has integrated clinical, analytical, and radiological variables with a neuropsychological assessment into a predictive model.

## 5. Conclusions

This study suggests that frailty, small-vessel cerebrovascular disease, and vitamin D deficiency are risk factors for depression after an episode of COVID-19 that requires hospital admission. For future research, we consider it useful to collect information from other hospitals and countries and perform an additional follow-up after 2 years for this diagnosis of chronic depression.

## Figures and Tables

**Table 1:** Characteristics of the patients included in the study.

Variable	*n* (%)
Sex	
Men	47 (65.3%)
Women	25 (34.5%)
Age (mean ± SD)	69.06 ± 8.70
Education level	
Not schooled	15 (20.8%)
Children’s school	16 (22.2%)
Elementary	20 (27.8%)
High school	16 (22.2%)
Higher education	5 (6.9%)
Hypertension	36 (50.0%)
DM2 ^1^	19 (26.4%)
COPD ^2^	6 (8.3%)
Asthma	8 (11.1%)
OSAHS ^3^	7 (9.7%)
Tobacco	7 (9.7%)
FRAIL scale	
Robust	13 (18.1%)
Pre-frail	40 (55.6%)
Frail	19 (26.4%)
Antidepressants	6 (8.3%)
BMI ^4^	29.47 ± 3.67
Charlson comorbidity index	1.46 ± 1.519
Days of admission	9.97 ± 6.959
Atrophy on MRI ^5^	46/69 (63.9%)
Cerebral small-vessel disease on MRI	28/69 (38.9%)
CAMCOG ^6^	
First visit	87.368 ± 11.038
Follow-up	90.354 ± 1.175
Vitamin D (ng/mL)	23.828 ± 13.504
CONUT ^7^	
First visit	4.177 ± 0.273
Follow-up at 6 months	0.985 ± 1.140
Frontal screening	
First visit	21.145 ± 4.241
Follow-up at 6 months	21.638 ± 0.514

Qualitative variables are expressed as a count (and percentage); quantitative variables are expressed as a mean ± standard deviation. ^1^ diabetes mellitus type 2; ^2^ COPD, chronic obstructive pulmonary disease; ^3^ obstructive sleep apnea/hypopnea syndrome; ^4^ body mass index; ^5^ magnetic resonance imaging; ^6^ Cambridge Cognitive Examination; ^7^ controlling nutritional status.

## Data Availability

The principal investigator, Sergio Salmerón, has all the study data.
